# Development of Type II Glucose Transporter Inhibitors: Phloretin as a GLUT-2 Screening Template from *In Silico* Modeling to *In Vitro* Assessment

**DOI:** 10.3390/biomedicines14051166

**Published:** 2026-05-21

**Authors:** Worarat Boonpech, Pemikar Srifa, Dhassida Sooksawat, Praopim Limsakul, Jirakrit Saetang, Varomyalin Tipmanee, Krit Charupanit, Chaitong Churuangsuk, Kantida Juncheed

**Affiliations:** 1 Institute of Biomedical Engineering, Faculty of Medicine, Prince of Songkla University, Hat Yai, Songkhla 90110, Thailand; worarat.bp43@gmail.com (W.B.);; 2 Department of Biomedical Sciences and Biomedical Engineering, Faculty of Medicine, Prince of Songkla University, Hat Yai, Songkhla 90110, Thailandtvaromya@medicine.psu.ac.th (V.T.); 3 Translational Medicine Research Center (TMRC), Department of Biomedical Sciences and Biomedical Engineering, Faculty of Medicine, Prince of Songkla University, Hat Yai, Songkhla 90110, Thailand; 4 Division of Physical Science, Faculty of Science, Prince of Songkla University, Hat Yai, Songkhla 90110, Thailand; 5 Center of Excellence for Trace Analysis and Biosensor, Prince of Songkla University, Hat Yai, Songkhla 90110, Thailand; 6 International Center of Excellence in Seafood Science and Innovation, Faculty of Agro-Industry, Prince of Songkla University, Hat Yai, Songkhla 90110, Thailand; jirakrit.s@psu.ac.th; 7 Center of Biological Activities Testing, Department of Biomedical Sciences and Biomedical Engineering, Faculty of Medicine, Prince of Songkla University, Hat Yai, Songkhla 90110, Thailand; chaitong.c@psu.ac.th; 8 Clinical Nutrition and Obesity Medicine Unit, Department of Internal Medicine, Faculty of Medicine, Prince of Songkla University, Hat Yai, Songkhla 90110, Thailand

**Keywords:** hepatocellular carcinoma, GLUT-2, phloretin, glucose uptake, molecular docking, cancer metabolism, GLUT inhibitors

## Abstract

**Background/Objectives:** Hepatocellular carcinoma (HCC) exhibits enhanced glycolytic activity, primarily facilitated by Class I glucose transporters (GLUTs), particularly GLUT-2. Phloretin, a natural polyphenol, is known to modulate glucose transport; however, its isoform-specific interactions and functional impact on HCC metabolism remain unclear. This study compared phloretin’s inhibitory effects on glucose uptake in HCC cells versus normal liver cell models and assessed its binding affinity across Class I GLUTs using molecular docking. **Methods:** Cytotoxicity was evaluated in HepG2 (HCC) and THLE-2 (normal hepatocyte) cells using 3-(4,5-Dimethylthiazol-2-yl)-2,5-diphenyltetrazolium bromide assays to determine biologically relevant concentrations. Glucose uptake at sub-cytotoxic levels was quantified using the fluorescent analog 2-(N-(7-Nitrobenz-2-oxa-1,3-diazol-4-yl)Amino)-2-Deoxyglucose. To elucidate the molecular mechanism, *in silico* docking simulations were performed to compare the binding affinities of phloretin, glucose, and reference inhibitors (glutor and cytochalasin B) with the outward-facing conformations of GLUT-1 through GLUT-4. **Results:** Phloretin induced dose- and time-dependent cytotoxicity, with HepG2 cells exhibiting significantly higher sensitivity than THLE-2 cells. Functionally, phloretin markedly reduced glucose uptake in HepG2 cells, whereas THLE-2 cells showed minimal inhibition. Molecular docking revealed that phloretin occupies the central substrate-binding cavity of Class I GLUTs, forming its most stable interaction network with GLUT-2. **Conclusions:** These results demonstrate that phloretin selectively inhibits glucose uptake in liver cancer cells, likely through its high-affinity interaction with GLUT-2. Collectively, these findings highlight phloretin’s potential as a metabolic therapeutic agent and support GLUT-2 as a viable target for HCC intervention.

## 1. Introduction

Hepatocellular carcinoma (HCC) is the second leading cause of cancer-related mortality globally [1,2]. A major clinical challenge is that most HCC cases are typically diagnosed at advanced, unresectable stages. Furthermore, the efficacy of curative interventions is severely hampered by critical limitations, including high rates of tumor recurrence, the scarcity of suitable donor organs for liver transplantation, and significant systemic cytotoxicity associated with conventional chemotherapy. Collectively, these factors contribute to poor patient prognosis and low overall survival [3,4]. Consequently, the development of effective, alternative therapeutic strategies is imperative to enhance long-term outcomes for patients with HCC [5,6].

Targeted therapies offer a promising alternative by specifically inhibiting molecular pathways critical for tumor progression, thereby avoiding the systemic toxicity of conventional chemotherapy [7]. This approach frequently targets the hallmark of metabolic reprogramming, which drives substantially increased glucose uptake in malignant cells compared to non-malignant tissue [8]. This metabolic alteration, known as the Warburg effect, is characterized by the shift from efficient oxidative phosphorylation to rapid, yet inefficient, aerobic glycolysis [9,10]. This heightened glycolytic dependence necessitates the upregulation of Class I glucose transporters (GLUTs), a common and clinically important feature observed in numerous malignancies, including HCC [10,11,12,13].

Among the Class I GLUTs, GLUT type II (GLUT-2), encoded by the Solute Carrier Family 2 Member 2 (*SLC2A2*) gene, is highly expressed in terminally differentiated hepatocytes [14,15]. Consistent with the Warburg effect, several studies have reported a marked overexpression of GLUT-2 in HCC tissue relative to normal liver parenchyma, suggesting its potential as a prognostic marker for the malignancy [14,16]. Such overexpression may contribute to the increased glucose uptake and enhanced glycolytic activity typical of rapidly proliferating tumor cells [17]. Based on these findings, therapeutic inhibition of GLUT-2 offers a viable targeted strategy to effectively deprive cancer cells of essential bioenergetic resources [18,19]. The specific role of GLUT-2 inhibition has been further highlighted as a key therapeutic direction for liver cancer [20]. Developing novel GLUT-2 inhibitors requires a rigorous *in vitro* screening cascade to evaluate their anticancer potential [21,22]. Importantly, for GLUT inhibitor assessment, the initial screening should also prioritize a glucose uptake inhibition assay to confirm their functional mechanism of action [23]. Initial efforts focus on identifying promising lead compounds, including naturally derived molecules such as phloretin, quercetin, myricetin, and fisetin, as well as other polyphenols, including resveratrol and naringenin [24,25]. Concurrently, synthetic agents with improved selectivity and potency have been developed, including the non-PPARγ thiazolidinedione derivative Compound 30 [26] and the highly specific G2iA and G2iB series, identified through *in silico* screening and functional validation [20].

For this development pipeline, establishing robust cellular models that provide an *in vivo*-like metabolic profile is mandatory. In this study, the natural dihydrochalcone phloretin is designated as an ideal reference compound and functional benchmark. This choice is strongly supported by extensive preclinical evidence demonstrating that phloretin-mediated glucose transport suppression inhibits cell growth, reduces metastatic potential, and induces apoptosis across multiple tumor models, including triple-negative breast and colon cancers [27,28,29].

In this study, molecular docking was utilized as a primary computational framework to characterize the binding interactions between Class I GLUT isoforms and a diverse panel of ligands, including the native substrate glucose, synthetic piperazine-based inhibitor glutor, plant-derived polyphenol phloretin, and fungal metabolite cytochalasin B. By elucidating these *in silico* binding modalities, we aimed to define the structural determinants of their varying inhibitory potencies. These insights positioned phloretin as a key structural template for exploring the development of plant-extracted GLUT-2 inhibitors. To address the current knowledge gap regarding phloretin’s selective binding and activity, we established a validated *in vitro* assay protocol to compare its inhibitory effects on HCC cells (HepG2) versus non-transformed hepatocytes (THLE-2). Within this experimental design, glutor served as a positive control for benchmarking glucose uptake inhibition [30]. Ultimately, this dual *in silico* and *in vitro* strategy provides a standardized platform for benchmarking GLUT-2 inhibition, validating phloretin as a foundational template for future structure-based GLUT-2 inhibitor development.

## 2. Materials and Methods

### 2.1. Cell Culture

HepG2 (human HCC; ATCC HB-8065; ATCC, Manassas, VA, USA) and THLE-2 (human immortalized normal hepatocyte; ATCC CRL-2706) cell lines were used as *in vitro* models for HCC and normal liver cells, respectively, and maintained under standard culture conditions. Briefly, HepG2 cells were cultured in Dulbecco’s Modified Eagle Medium (DMEM; Gibco, Thermo Fisher Scientific, Waltham, MA, USA; Cat. No. 11995-065) supplemented with 10% fetal bovine serum (FBS; Gibco, Thermo Fisher Scientific, Waltham, MA, USA; Cat. No. A5209401) and 1% penicillin–streptomycin (Gibco, Thermo Fisher Scientific, Waltham, MA, USA; Cat. No. 15140-122). THLE-2 cells were cultured in Bronchial Epithelial Cell Growth Medium (BEGM; Lonza, Basel, Switzerland; Cat. No. CC-3171) supplemented with the BEGM SingleQuots™ Kit (Lonza, Basel, Switzerland; Cat. No. CC-4175), containing growth factors, insulin, transferrin, and epidermal growth factor (EGF; Gibco, Thermo Fisher Scientific, Waltham, MA, USA; Cat. No. PHG0311), according to ATCC recommendations. All cells were incubated at 37 °C in a humidified atmosphere containing 5% CO_2_ (Model: SCO6AD-2; SHEL LAB, Cornelius, OR, USA), with medium replaced every 2–3 days to maintain exponential growth until reaching 70–80% confluency before experimentation. Additionally, HepG2 and THLE-2 cell lines were routinely monitored for mycoplasma contamination via high-magnification light microscopy. No evidence of contamination, such as altered growth kinetics or cytoplasmic granulation, was observed during the experimental period.

### 2.2. Cytotoxic Assessment of Phloretin

Cell viability following phloretin treatment was quantified using the 3-(4,5-Dimethylthiazol-2-yl)-2,5-diphenyltetrazolium bromide (MTT) assay (EMD Millipore, Burlington, MA, USA; Cat. No. CT01) in both cell types. Briefly, HepG2 and THLE-2 cells were seeded at 1 × 10^4^ cells/well in 96-well plates and allowed to adhere overnight. Subsequently, cells were treated with phloretin (Sigma-Aldrich, St. Louis, MO, USA; Cat. No. P7912) 0–800 µM for 24 or 48 h. After treatment, 10 µL of 5 mg/mL MTT solution was added to each well and incubated for 4 h. Formazan crystals were dissolved in 100 µL dimethyl sulfoxide (DMSO; Sigma-Aldrich, St. Louis, MO, USA; Cat. No. BCCH3300), and absorbance was measured at 570 nm using a microplate reader (Infinite M Plex; Tecan, Männedorf, Switzerland). The half-maximal inhibitory concentration (IC_50_) was defined as the concentration of phloretin that reduces cellular metabolic activity by 50%, reflecting a corresponding decrease in cell viability and proliferation. Two independent experiments were conducted, each performed in triplicate wells for every condition. Cellular morphology was examined under an inverted phase-contrast microscope (Primovert; Carl Zeiss, Oberkochen, Germany) to assess structural changes induced by phloretin treatment.

### 2.3. Glucose Uptake Assay

Cellular glucose uptake was assessed using 2-(N-(7-Nitrobenz-2-oxa-1,3-diazol-4-yl)Amino)-2-Deoxyglucose (2-NBDG) (Sigma-Aldrich, St. Louis, MO, USA; Cat. No. 72987), a fluorescent non-metabolizable glucose analog. Before application in comparative assays, incubation time was optimized to balance signal intensity and cell adherence. HepG2 cells were exposed to 100 µM 2-NBDG for 30, 45, 60, or 120 min, and fluorescence was measured to identify the optimal interval. The 45 min condition was selected for subsequent experiments as it yielded a strong signal with minimal monolayer disruption as shown in Supplementary Materials, Figure S1. For inhibitory assays, HepG2 and THLE-2 cells were pretreated with phloretin at 0.5 and 1.0 IC_50_ concentrations for 24 h, followed by incubation with 100 µM 2-NBDG in glucose-free DMEM medium (Corning, Corning, NY, USA; Cat. No. 17-207-CV) for 45 min. and fluorescence intensity (FI) was measured (excitation 465 nm and emission 540 nm). Representative fluorescence images were acquired using a fluorescence microscope (Lionheart™ FX Live Cell Imager; BioTek Instruments, Winooski, VT, USA). to confirm uptake visually.

### 2.4. Molecular Docking

Predicted structural models of all Class I GLUT isoforms—GLUT-1 (UniProtID: P11166), GLUT-2 (UniProtID: P11168), GLUT-3 (UniProtID: P11169), and GLUT-4 (UniProtID: P14672)—were generated using AlphaFold structural predictions as the initial backbone. Each model was further refined using SWISS-MODEL, applying the outward-facing crystal structure of human GLUT-3 (PDB ID: 4ZWC.1.A) as a common template to ensure conformational consistency across isoforms. Model quality and stereochemical reliability were assessed using Global Model Quality Estimation and Qualitative Model Energy Analysis scoring metrics. Ligand structures were retrieved from the PubChem database, including glucose (CID: 5793), glutor (CID: 166419415), cytochalasin B (CID: 5311281), and phloretin (CID: 4788). Each ligand was geometry optimized using Gaussian 09 at the B3LYP/6-31G (d,p) [31] level of theory before docking to ensure accurate electronic and structural representation for binding analysis.

Docking simulations were conducted using AutoDock 4.2 (The Scripps Research Institute, La Jolla, CA, USA) with a Lamarckian genetic algorithm. A grid box of 25 × 25 × 25 Å^3^ (equivalent to 68 × 68 × 68 grid points at 0.375 Å spacing) was used to fully enclose the central pore and capture potential ligand conformations. Additionally, 50 independent GA runs were performed with a population size of 200. The docking score, defined by the predicted binding free energy (kcal/mol), was used to rank ligand conformations. The lowest-energy binding poses were selected for further analysis. Protein–ligand interactions were visualized in Discovery Studio 2024 (version 4.5; BIOVIA, San Diego, CA, USA) [32], focusing on hydrogen bonding, hydrophobic interactions, and π–π stacking to characterize the molecular basis of binding.

### 2.5. Statistical Analysis

All data are presented as mean ± standard deviation (SD) from at least three independent experiments. Data normality was assessed using the Shapiro–Wilk test in GraphPad Prism (version 10.6.1; GraphPad Software, San Diego, CA, USA), and all datasets satisfied the normality assumption (*p* > 0.05); therefore, parametric tests were applied. For experiments involving a single factor, statistical comparisons were performed using one-way analysis of variance (ANOVA) followed by Dunnett’s post hoc test to compare treatment groups with the control group, whereas for experiments involving two independent factors, statistical comparisons were performed using two-way ANOVA to evaluate main effects and their interaction, followed by Tukey’s multiple comparisons test. Statistical significance was set at *p* < 0.05, and IC_50_ values were calculated using nonlinear regression analysis in GraphPad Prism.

## 3. Results

### 3.1. In Silico Study of Glucose Uptake Inhibition

**Figure 1:**
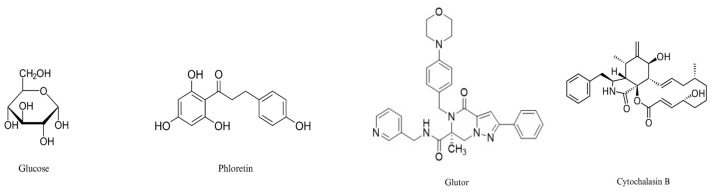
Chemical structures of glucose, phloretin, glutor, and cytochalasin B.

Glucose, the native substrate of Class I GLUTs, was first docked into all GLUT isoforms (GLUT-1 to GLUT-4) in the outward-facing conformation to establish a structural reference for the analysis. The resulting binding poses and docking scores provided a baseline for comparison and enabled the visualization of conserved features within the extracellular substrate-binding cavity across isoforms. Glucose and the selected inhibitors, including phloretin, glutor, and cytochalasin B, were chosen to evaluate the docking dynamics of Class I GLUTs using a diverse chemical library. Glucose served as the essential native substrate control, while the other inhibitors represented distinct pharmacological origins: glutor as a potent synthetic piperazine-based inhibitor, phloretin as a plant-derived natural dihydrochalcone, and cytochalasin B as a classic fungal-derived mycotoxin. These ligands were further prioritized based on their favorable solubility and stability profiles, as well as their varied chemical structures, enabling a comprehensive analysis of the transporter’s binding pocket across different structural molecules. Using the same outward-facing models and identical docking parameters, inhibitory ligands (phloretin, glutor, and cytochalasin B) were subsequently docked to compare inhibitor binding with glucose (Figure 1). This approach enabled systematic evaluation of ligand placement, binding orientation, residue-level interactions, and relative docking scores within the shared substrate entry site features that cannot be directly observed *in vitro*. This computational component delivers structural insights that support the biological data and clarify the observed affinity of phloretin toward GLUT-2.

**Figure 2:**
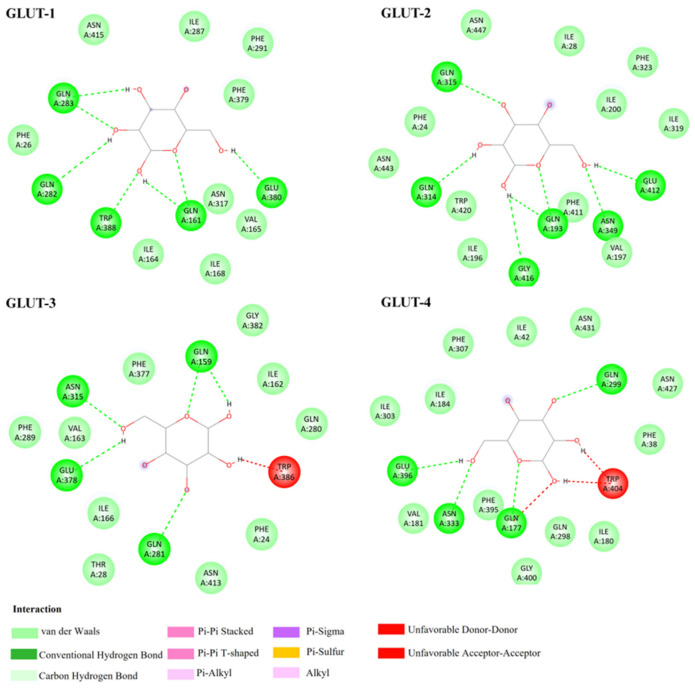
Predicted binding interactions of glucose with Class I GLUT isoforms. Two-dimensional interaction diagrams showing the binding mode of glucose within the substrate cavity of GLUT-1, -2, -3, and -4. (GLUT, glucose transporter). 2D interaction diagrams were generated using BIOVIA Discovery Studio Visualizer.

First, docking analysis of glucose with Class I GLUTs (GLUT-1–GLUT-4) revealed comparable binding scores ranging from −4.3 to −4.7 kcal/mol (Table 1), consistent with its role as the shared physiological substrate of these isoforms. Although GLUT-2 demonstrated the lowest binding energy among the isoforms, the overall docking scores across Class I GLUTs did not differ substantially. This uniformity reflects the conserved architecture of the glucose-binding pocket among Class I GLUTs, which share homologous transmembrane helices and substrate-coordinating residues. Figure 2 illustrates the predicted molecular interactions between glucose and each isoform. In all models, glucose binding was primarily stabilized through hydrogen bonds with conserved polar residues, including GLN, ASN, and GLU. Isoform-specific differences were also apparent. GLUT-3 and GLUT-4 showed unfavorable contacts or mild steric interference involving TRP residues, whereas GLUT-2 displayed additional stabilization through a hydrogen bond with a GLY residue located in the substrate-binding region. These subtle variations highlight both the shared substrate-recognition framework and the minor structural nuances distinguishing individual GLUT isoforms.

**Figure 3:**
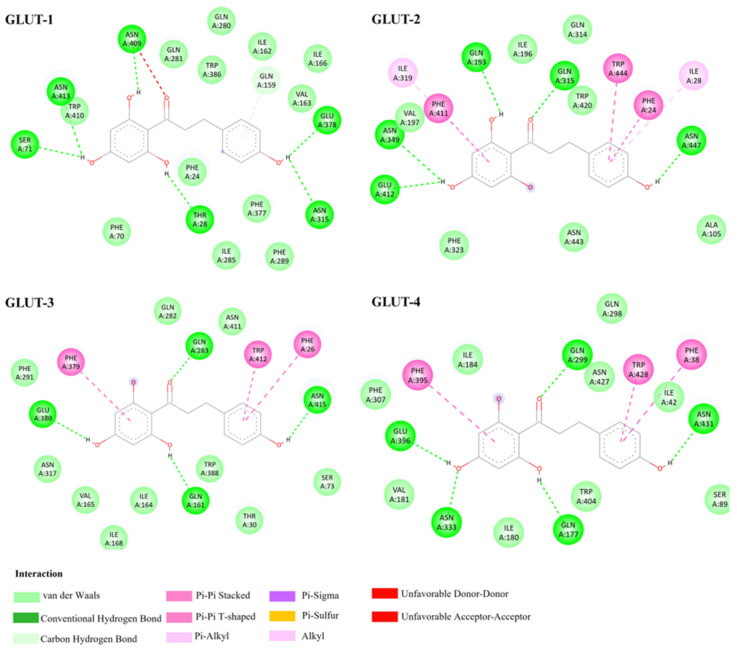
Predicted binding interactions of phloretin with Class I GLUT isoforms. Two-dimensional interaction diagrams showing the binding mode of phloretin within the substrate cavity of GLUT-1, GLUT-2, GLUT-3, and GLUT-4. 2D interaction diagrams were generated using BIOVIA Discovery Studio Visualizer.

Second, docking analysis revealed that phloretin exhibited moderately negative binding free energies across all Class I GLUT isoforms, ranging from −6.9 to −7.5 kcal/mol (Table 1), indicating a higher predicted affinity than glucose. Despite minor energetic differences, phloretin displayed a consistent binding pattern across GLUT-1, GLUT-2, GLUT-3, and GLUT-4 aligning parallel to the transmembrane between TM7 and TM10. In all isoforms, stabilization was primarily mediated through hydrogen bonding to conserved polar residues and aromatic or π-driven interactions involving TRP and PHE residues, reflecting a shared substrate recognition framework among Class I GLUTs, as shown in Figure 3. Isoform-specific differences became apparent when examining residue-level interactions. GLUT-1, GLUT-3, and GLUT-4 primarily interacted with phloretin through polar amino acids, including GLN, ASN, and GLU, resulting in broadly similar docking orientations with only subtle variations in binding strength. Notably, in GLUT-3, an unfavorable interaction was detected at ASN409. This unfavorable contact distinguishes GLUT-3 from the other isoforms and may partially explain its slightly weaker interaction profile than GLUT-4. In contrast, GLUT-2 displayed the most extensive interaction network, forming hydrogen bonds with GLN193, GLN315, ASN349, GLU412, and ASN447, together with π–π interactions involving PHE24, PHE415, and TRP444. Additionally, π–alkyl interactions with ILE133 and LEU135 were observed exclusively in GLUT-2 and not in other Class I isoforms. This comprehensive interaction profile suggests that GLUT-2 offers a more accommodating and hydrophobic binding pocket for phloretin, consistent with its slightly lower binding energy of −6.96 kcal/mol. These findings highlight GLUT-2 as the isoform most capable of forming a stable phloretin transporter complex, a characteristic that may be advantageous for the future development of selective GLUT-2 inhibitors.

**Figure 4:**
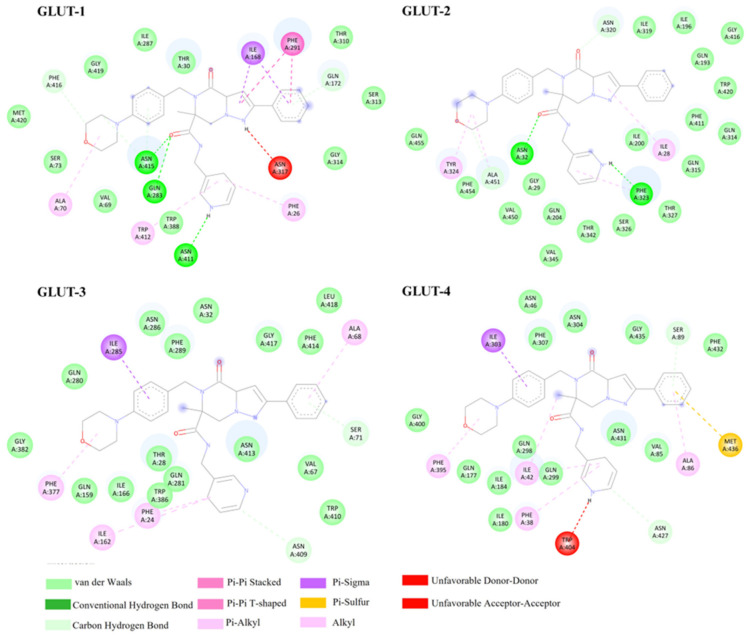
Predicted binding interactions of glutor with Class I GLUT isoforms. Two-dimensional interaction diagrams showing the binding mode of glutor within the substrate cavity of GLUT-1, -2, -3, and -4. 2D interaction diagrams were generated using BIOVIA Discovery Studio Visualizer.

Third, glutor showed the most negative binding free energies among all tested ligands, with values ranging from −9.43 to −10.94 kcal/mol, indicating exceptionally strong predicted affinity toward Class I GLUTs (Table 1). Across all isoforms, glutor occupied the central transmembrane cavity and formed a consistent pattern of stabilizing interactions, including hydrogen bonds, carbon–hydrogen bonds, π–alkyl contacts, π–π stacking, and extensive van der Waals contacts. These shared features reflect a conserved inhibitory mode of binding across GLUT-1, GLUT-2, GLUT-3, and GLUT-4. Isoform-specific interaction patterns became clear upon examining the residue contacts shown in Figure 4. In GLUT-2, the ligand established hydrogen-bond interactions with ASN32 and PHE323, together with carbon–hydrogen bonds involving ALA451 and ASN320, and multiple π–alkyl interactions with ILE28 and TYR324. These contacts were complemented by extensive van der Waals interactions from surrounding hydrophobic residues, collectively creating a densely packed binding cavity. This configuration produced the most binding energy (−10.94 kcal/mol) observed among all Class I GLUTs. In comparison, GLUT-1 and GLUT-3 showed fewer hydrophobic contacts and relied more on aromatic and alkyl stabilization, including π–π stacking with PHE291 (GLUT-1) and π–alkyl interactions with residues such as PHE26 (GLUT-1), TRP412 (GLUT-1), ILE162 (GLUT-3), and ALA68 (GLUT-3). Both isoforms also displayed π–sigma interactions, specifically involving ILE168 (GLUT-1) and ILE285 (GLUT-3), further supporting aromatic-aliphatic stabilization in these complexes. GLUT-4 displayed a distinctive π–sulfur interaction with MET436, a feature absent in other isoforms, along with additional π–alkyl contacts involving ILE42, PHE38, and PHE395, and a π–sigma interaction with ILE303. Overall, glutor’s broad and diverse interaction network—including π–sigma and π–sulfur, along with pronounced steric interference from surrounding hydrophobic residues—supports its strong predicted affinity and known role as a potent pan-GLUT inhibitor.

**Figure 5:**
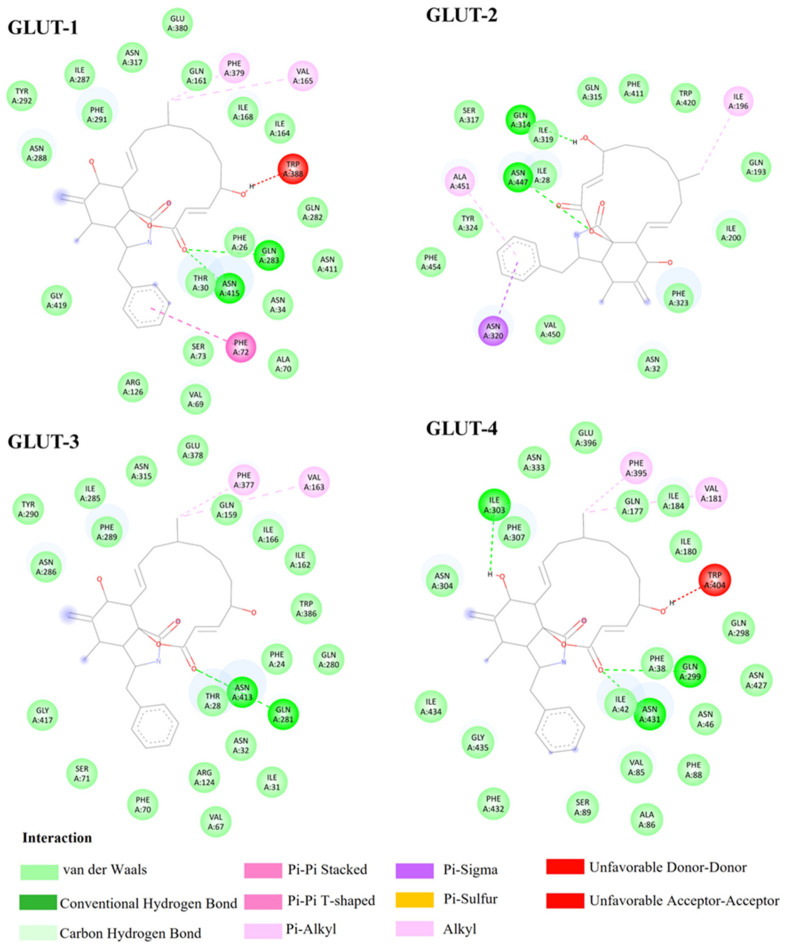
Predicted binding interactions of cytochalasin B with Class I GLUT isoforms. Two-dimensional interaction diagrams showing the binding mode of cytochalasin B within the substrate cavity of GLUT-1, GLUT-2, GLUT-3, and GLUT-4. 2D interaction diagrams were generated using BIOVIA Discovery Studio Visualizer.

Finally, cytochalasin B exhibited consistently strong binding affinities across all Class I GLUTs, with docking scores ranging from −8.90 to −9.69 kcal/mol (Table 1). A shared interaction pattern was observed in all isoforms, including the formation of conventional hydrogen bonds with conserved polar residues, π–alkyl and π–sigma interactions with hydrophobic and aromatic side chains, and extensive van der Waals contacts (Figure 5). In GLUT-1, cytochalasin B established hydrogen bonds with ASN415 and GLN283, together with π–π stacking and π–alkyl interactions involving PHE72, PHE379, and VAL165. A mild unfavorable interaction was detected at TRP388. For GLUT-3, stabilization occurred through hydrogen bonding with ASN413, carbon–hydrogen bonding with ASN413 and GLN281, and π–alkyl contacts with VAL163 and PHE377, supported by extensive van der Waals interactions. In GLUT-4, cytochalasin B formed hydrogen bonds with ILE303, GLN299, and ASN431, along with π–alkyl interactions involving PHE395 and VAL181. An unfavorable interaction was observed at TRP404. Conversely, GLUT-2 presented hydrogen bonds with ASN320 and GLN314, along with a π–sigma interaction at ASN320 and π–alkyl contacts with ILE196 and ALA451, resulting in the most favorable binding energy (−9.69 kcal/mol). Overall, cytochalasin B demonstrated a consistent combination of hydrogen bonding, π-driven interactions, and hydrophobic contacts across all Class I GLUTs, corresponding with the strong binding affinities predicted in this simulation.

Based on Section 4.1 (*in silico* analysis of selected GLUT-2 inhibitors), phloretin was selected over higher-affinity synthetic ligands like glutor and cytochalasin B due to its superior GLUT-2 selectivity and more favorable safety profile. Consequently, phloretin was employed as the lead compound to further evaluate its biological impact through cytotoxicity and glucose uptake assays. These subsequent experiments aim to validate the computational predictions by characterizing its functional efficacy and safety in a cellular environment.

### 3.2. Cytotoxicity of Phloretin in HepG2 and THLE-2 Cell Lines

**Figure 6:**
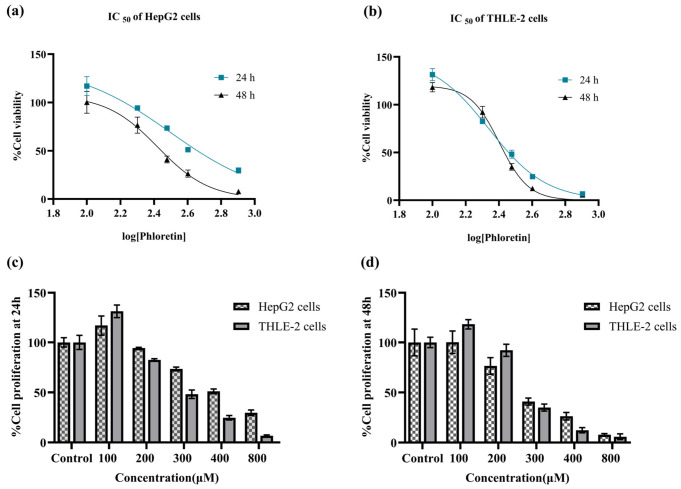
Cytotoxic effects of phloretin on HepG2 and THLE-2 cells. (**a**,**b**) Dose–response curves in HepG2 and THLE-2 cells after 24 and 48 h treatment. (**c**,**d**) Bar graphs showing dose-dependent reduction in proliferation across time points. Data are presented as mean ± SD (*n* = 3). Data are presented as mean ± SD from at least three independent experiments. Statistical analysis was performed using one-way ANOVA followed by Dunnett’s test.

**Figure 7:**
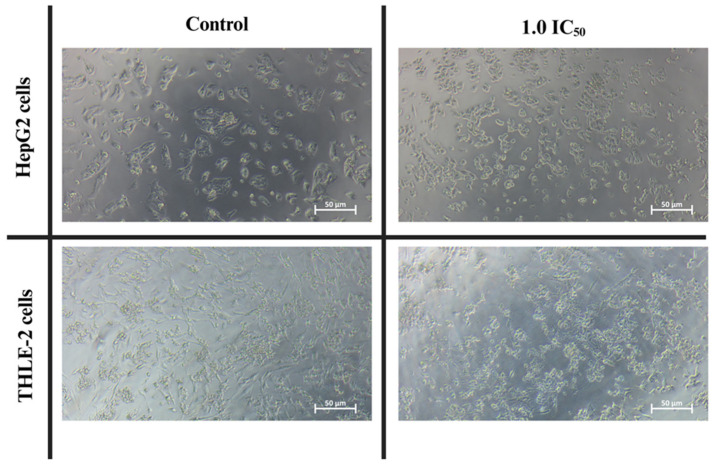
Morphological changes in HepG2 and THLE-2 cells after phloretin treatment. Representative phase-contrast images of HepG2 and THLE-2 cells after 24 h exposure to control medium or phloretin at the IC_50_ concentration. Images were acquired using an inverted light microscope (Lionheart FX, BioTek Instruments, Winooski, VT, USA) at 10× magnification. Scale bar = 50 µm.

Cytotoxicity assessment was conducted to identify biologically relevant, non-lethal concentrations of phloretin for subsequent functional assays. To screen the effects of phloretin on cellular metabolism, an MTT assay was performed on HepG2 and THLE-2 cell lines across a concentration range of 100 to 800 µM for 24 h. The resulting dose–response profiles, provided in Supplementary Materials, Figure S2, demonstrate a reduction in metabolic activity as concentration increases. Given that phloretin exhibits antiproliferative activity, IC_50_ values at 24 and 48 h were determined to distinguish direct effects on glucose uptake from nonspecific cytotoxicity and guide dose selection for downstream experiments. As shown in Figure 6, phloretin treatment led to a significant, dose-dependent reduction in metabolic activity in both cell lines. At 24 h, the IC_50_ values were 315 ± 46 µM and 228 ± 11 µM for HepG2 and THLE-2, respectively. At 48 h, HepG2 cells showed increased sensitivity with an IC_50_ of 268 ± 13 µM, whereas THLE-2 cells exhibited partial adaptation with an IC_50_ of 243 ± 9.7 µM. Morphological observations at 24 h, as illustrated in Figure 7, further supported the cytotoxicity data. Cells treated with phloretin at the 24 h IC_50_ concentrations exhibited reduced cell density and adherence, along with noticeable cell shrinkage compared with untreated controls. Overall, HepG2 cells demonstrated progressive vulnerability to phloretin over time, while THLE-2 cells showed higher initial sensitivity but partial recovery with prolonged exposure. These findings indicate distinct cellular responses to GLUT-2 inhibition between malignant HCC cells and +normal hepatocytes.

### 3.3. Effect of Phloretin on Glucose Uptake in HepG2 and THLE-2 Cell Lines

**Figure 8:**
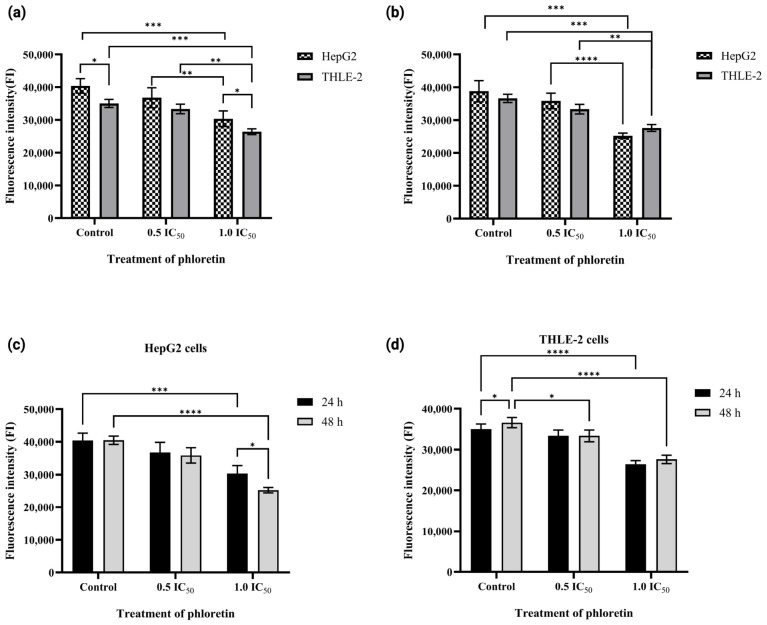
Effect of phloretin on glucose uptake in HepG2 and THLE-2 cells. (**a**,**b**) Relative fluorescence intensity (FI) of 2-NBDG uptake after (**a**) 24 h and (**b**) 48 h, normalized to time-matched controls. (**c**,**d**) Time-dependent comparison normalized to the 24 h control, showing progressive inhibition and stable uptake in HepG2 and THLE-2 cells, respectively. Data are presented as mean ± SD (*n* = 3). Statistical analysis was performed using two-way ANOVA followed by Tukey’s post hoc test. Asterisks above bars indicate significant differences vs. the corresponding control (* *p* < 0.05, ** *p* < 0.01, *** *p* < 0.001, **** *p* < 0.0001), and asterisks between bars denote significant differences between time points for the same treatment.

Glucose uptake assays were performed following IC_50_ determination, thereby enabling the assessment of phloretin’s functional effect on cellular glucose transport and its ability to inhibit glucose availability at the transporter level. Glucose uptake was measured using the fluorescent glucose analog 2-NBDG in HepG2 and THLE-2 cells to compare the inhibitory response. Phloretin significantly inhibited glucose uptake in HepG2 cells in a dose- and time-dependent manner (Figure 8). After 24 h of treatment, the FI of 2-NBDG uptake decreased progressively with increasing phloretin concentrations compared to untreated controls (*p* < 0.05). The inhibitory effect became more pronounced after 48 h, with FI values declining to approximately 50% of control. In contrast, THLE-2 cells exhibited only modest reductions in fluorescence at equivalent concentrations, with no significant time-dependent variation, indicating relative resistance to phloretin-induced transport inhibition compared with HepG2.

**Figure 9:**
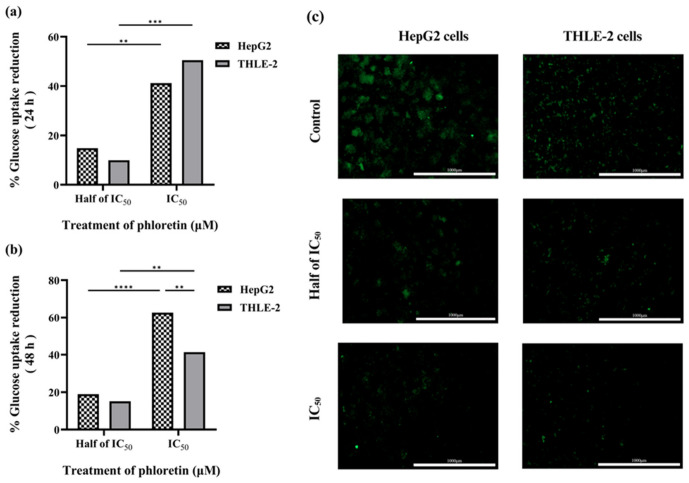
Inhibitory effect of phloretin on glucose uptake in HepG2 and THLE-2 cells. (**a**,**b**) Percentage reduction in 2-NBDG uptake after (**a**) 24 h and (**b**) 48 h of phloretin exposure at 0.5 and 1.0 IC_50_, relative to control. (**c**) Representative fluorescence images showing decreased 2-NBDG fluorescence intensity in HepG2 and minimal changes in THLE-2. Data are presented as mean ± SD (*n* = 3). Statistical analysis was performed using two-way ANOVA with Tukey’s post hoc test. ** *p* < 0.01, *** *p* < 0.001, **** *p* < 0.0001, vs. Control. Scale bar = 1000 µm.

As shown in Figure 9, the mean percentage reduction in glucose uptake in HepG2 cells was approximately 15% and 41% at 0.5 and 1.0 IC_50_, respectively, after 24 h, increasing to approximately 19% and 63% after 48 h. In contrast, THLE-2 cells showed smaller decreases of approximately 10% and 50% after 24 h and 15% and 41% after 48 h, respectively, under the same conditions. Overall, these results demonstrate that phloretin selectively and progressively suppresses glucose uptake in HepG2 cells, consistent with GLUT-2-mediated inhibition, while THLE-2 cells remain relatively resistant to phloretin-induced transport blockade.

## 4. Discussion

### 4.1. In Silico Analysis of Selected GLUT-2 Inhibitors

Understanding the molecular interactions between candidate inhibitors and the GLUT-2 transporter is essential for the rational design of targeted therapies for HCC. GLUT-2 (SLC2A2), a low-affinity, high-capacity GLUT, plays a vital role in hepatic glucose sensing and systemic glucose homeostasis [33]. In HCC, GLUT-2 is frequently overexpressed, contributing to elevated glucose uptake and supporting rapid tumor proliferation [2]. In this study, molecular docking analysis was employed to characterize the binding affinities and interaction mechanisms of phloretin, glucose, and synthetic ligands, including glutor and cytochalasin B. As, phloretin, glutor, and cytochalasin B are distinct chemical scaffolds like natural dihydrochalcone, synthetic piperazine-based inhibitor, and fungal macrocyclic lactone, respectively and each interacts with the GLUT-2 substrate cavity through different spatial configurations.

Phloretin, a naturally occurring dihydrochalcone [34], demonstrated a stronger binding affinity to GLUT-2 (−6.96 kcal/mol) than glucose (−4.68 kcal/mol). Moreover, analysis of the ligand–receptor interactions demonstrates that glucose and phloretin occupy a shared binding site within the GLUT-2 pocket, involving key residues such as GLN193, GLN314, GLN315, GLU412, ASN443, ASN447, ILE196, ILE200, ILE319, PHE323, PHE411, TRP420, VAL197, ASN349, and GLY416. The high degree of contact overlap between the two molecules provides strong evidence for direct competitive inhibition. Furthermore, phloretin forms unique π–π T-shaped and π–alkyl interactions with PHE24, TRP444, and ILE28 these contacts are absent in the glucose-bound complex. These specific aromatic and hydrophobic interactions likely enhance the stabilization of phloretin within the site, reinforcing its efficacy as a potent GLUT-2 inhibitor, suggesting competitive inhibition at the canonical substrate-binding site, see Supplementary Materials, Figure S3. The docking pose of phloretin indicated stabilizing hydrogen bonds and π–π stacking interactions with conserved amino acid residues, contributing to enhanced binding stability. The evaluation of interaction profiles (Table 1) revealed that ligands capable of forming π-mediated interactions—particularly π–π stacking and π–alkyl contacts—were generally associated with greater binding stability than those relying solely on hydrogen bonding. Although hydrogen bonds help orient ligands within the substrate cavity, they are insufficient to maintain high-affinity binding without hydrophobic reinforcement. Moreover, steric hindrance due to spatial clashes negatively affected docking scores, even when polar interactions were present. These findings suggest that π-mediated hydrophobic interactions are the dominant stabilizing force, with spatial compatibility within the GLUT-2 pocket playing a critical role. Although synthetic ligands, including glutor and cytochalasin B, demonstrated stronger docking scores (−10.94 and −9.69 kcal/mol, respectively), they showed broader activity across GLUT isoforms, indicating lower selectivity for GLUT-2 and potential off-target effects.

Phloretin distinguishes itself not only through a favorable binding profile but also through practical, mechanistic, and safety-related advantages. As a plant-derived compound that can be extracted using ethanol, phloretin offers a more sustainable and less toxic alternative [35] than agents, including cytochalasin B, which require extraction with methanol, a solvent known to pose greater health and environmental risks [36]. Structurally, phloretin’s dual-ring aromatic system enables both polar and hydrophobic interactions, allowing it to anchor deeply within the GLUT-2 cavity [37]. Importantly, live-cell imaging studies have demonstrated that phloretin disrupts GLUT-2 membrane trafficking in polarized epithelial cells, suggesting an extended mechanism of inhibition beyond simple substrate competition [38]. This trafficking inhibition, in conjunction with competitive binding, may potentiate the suppression of glucose uptake in GLUT-2-dependent cancer cells.

Although phloretin does not exhibit the strongest binding affinity among the tested ligands, its intermediate yet consistent docking performance, engagement with multiple stabilizing interactions, and suppression of glucose uptake in GLUT-2 overexpressing cells justify its use as a reference compound. Furthermore, its natural origin and safety profile support its utility in early-stage screening and lead optimization. These findings support the development of phloretin-derived scaffolds and analogs as promising candidates for GLUT-2-targeted therapy [28,34,37]. Building on this molecular rationale, we subsequently evaluated whether phloretin’s predicted GLUT-2 interactions translated into functional inhibition of glucose uptake and metabolic suppression in cellular models.

### 4.2. Inhibitory Effect of Phloretin

GLUT-2 presents a structurally distinct substrate-binding landscape, with greater hydrophobic accessibility than other Class I GLUT isoforms, due to distinct amino acid substitutions that reduce steric hindrance and increase hydrophobic volume. Residues, including Ile29, Ile133, Leu135, and Ala198, form a broad internal loop that enlarges the pore, creating an open, flexible pocket compared with the narrower, more aromatic vestibules of GLUT-1, -3, and -4 [39,40]. The structurally accessible and hydrophobic binding cavity of GLUT-2 may facilitate phloretin accommodation within the transporter channel [33,41].

Our docking simulations supported these structural insights and revealed phloretin’s competitive binding within the GLUT-2 substrate pocket. Phloretin exhibited a more negative docking energy (−6.96 kcal/mol) than glucose (−4.68 kcal/mol), suggesting that phloretin may compete effectively with glucose for the same binding site [42]. Binding stability was supported by both hydrogen bonding and hydrophobic interactions within the GLUT-2 cavity. In contrast, the narrower and more polar substrate pockets of GLUT-1, -3, and -4, dominated by GLU and ASN residues [39,43,44], may limit hydrophobic interactions with phloretin, resulting in less stable binding.

This *in silico* evidence aligned with the *in vitro* findings, where phloretin induced dose- and time-dependent cytotoxicity, with HepG2 cells exhibiting greater sensitivity than THLE-2 cells over 48 h. This differential response reflects the metabolic reprogramming (Warburg effect) in HCC, in which cancer cells rely heavily on glucose uptake and glycolysis for survival [6,45]. In contrast, normal hepatocytes (THLE-2) showed comparatively lower sensitivity to phloretin treatment. This selective sensitivity suggests a potential therapeutic window in which cancer cells are preferentially affected by GLUT inhibition.

Consistent with the docking analysis, the optimized 45 min 2-NBDG uptake assay demonstrated that phloretin significantly reduced glucose uptake in HepG2 cells, whereas THLE-2 cells showed a comparatively weaker response. HepG2 cells demonstrated both a higher baseline 2-NBDG uptake and a more pronounced inhibition by phloretin than THLE-2. The stronger inhibitory effect of phloretin in HepG2 cells aligned with its higher cytotoxicity, suggesting greater metabolic vulnerability of HCC cells to GLUT inhibition. These findings are consistent with those of prior reports showing that blockade of GLUT-2 function reduces cancer cell viability: phloretin has been shown to inhibit glucose transport and growth in colorectal cancer and induce apoptosis in liver cancer models through GLUT-2 inhibition. Overall, the cytotoxicity and glucose uptake data support the potential of GLUT-targeted metabolic inhibition in HCC cells while exerting comparatively lower effects on normal liver cells.

Nevertheless, the IC_50_ values observed for phloretin in this study (228–315 µM) warrant careful consideration regarding clinical translatability. However, these values are comparable to several established GLUT inhibitors that also operate in the micromolar range such as fasentin and WZB117 typically demonstrate potencies in the 10–100 µM range [46,47,48]. Furthermore, while phloretin exhibits lower overall potency than synthetic agents like STF-31, it is noteworthy that the latter also shows significantly reduced efficacy (>100 µM) when tested in non-sensitized cell models [49,50]. Rather than a finalized clinical candidate, phloretin serves as a natural molecular scaffold. Our *in silico* data reveals that its dual-ring aromatic system provides a structural blueprint for future medicinal chemistry. By utilizing this interaction profile as a template, subsequent modifications aimed at enhancing binding affinity and isoform selectivity can transition this scaffold into high-potency analogs. Consequently, this study establishes the molecular foundation necessary to bridge the gap between natural product characterization and the development of clinically viable GLUT-2 inhibitors.

Despite the encouraging results, some limitations should be acknowledged. First, our experiments were confined to *in vitro* cell models. Although THLE-2 cells serve as a normal hepatocyte comparison, they are immortalized and may not fully recapitulate the behavior of primary hepatocytes in vivo. The true therapeutic index of phloretin requires evaluation in animal models of HCC, where factors including drug metabolism, tumor microenvironment, and systemic toxicity play critical roles. Notably, normal tissues—including the liver, kidney, and pancreas [51,52,53]—naturally express GLUT-2 for physiological glucose handling; therefore, systemic inhibition of GLUT-2 could disrupt normal glucose homeostasis. Second, phloretin lacks GLUT isoform specificity [54]. Phloretin is a broad-spectrum inhibitor that affects multiple GLUT isoforms (GLUT-1 to GLUT-4) at similar concentrations [42]. It can also interact with other membrane transporters (e.g., the vitamin C transporter SVCT1, as observed with related flavonoids) [55]. Moreover, as a lipophilic planar polyphenol, phloretin can insert into lipid membranes and alter their biophysical properties by reducing the membrane dipole potential, possibly triggering nonspecific signaling effects [56]. Such off-target and pleiotropic actions imply that not all observed antiproliferative effects in HepG2 cells can be attributed solely to GLUT-2 inhibition. For instance, phloretin may modulate signaling cascades (e.g., STAT3, Akt) [57,58,59] or induce oxidative stress independently of glucose transport [60]. These factors, together with the *in silico* predictions that still require experimental validation, urge caution in interpreting phloretin’s mechanism of action. In summary, although phloretin served as a reference GLUT-2 inhibitor, its broad activity and the *in vitro* nature of this study limit the immediate translational implications.

### 4.3. The Mechanistic Role of Phloretin in Targeting Glucose Metabolism and Inducing Apoptosis in HCC

The examination of natural compounds, particularly phloretin, for their ability to regulate cancer cell metabolism and trigger apoptosis represents a key aspect of oncological research. This study proposes an expected metabolic pathway through which phloretin suppresses glucose transport, indirectly initiating apoptotic events in HCC cells. The HepG2 cell line, a common *in vitro* model for HCC, expresses multiple GLUT isoforms, notably GLUT-2, which serves as a significant conduit for glucose transport in hepatic cells [16]. This high GLUT-2 expression is particularly relevant as clinical HCC specimens frequently exhibit a markedly elevated level of GLUT-2 compared to adjacent normal liver tissue [61]. Phloretin is acknowledged for its inhibitory effect on glucose transport across cellular membranes [34]. Therefore, the expected mechanism underlying phloretin’s action is hypothesized to involve the direct inhibition of these GLUT proteins. Our findings provide structural and functional evidence supporting this hypothesis. Specifically, computational simulations, including molecular docking analysis of phloretin and GLUT-2, demonstrate that phloretin likely binds to GLUT-2 in a manner competitive with glucose, thereby impeding glucose translocation into the intracellular space. This result is corroborated by existing literature, notably the study of Elmetwalli et al., which utilized *in silico* docking to confirm the potential competitive binding of phloretin as a GLUT-2 targeting agent [37]. Functionally, this blockade of GLUT-2 by phloretin was directly observed in our experimental studies, which demonstrated a measurable reduction in glucose uptake into the cancer cells via the 2-NBDG uptake assay as well as the observation of membrane shrinkage.

**Figure 10:**
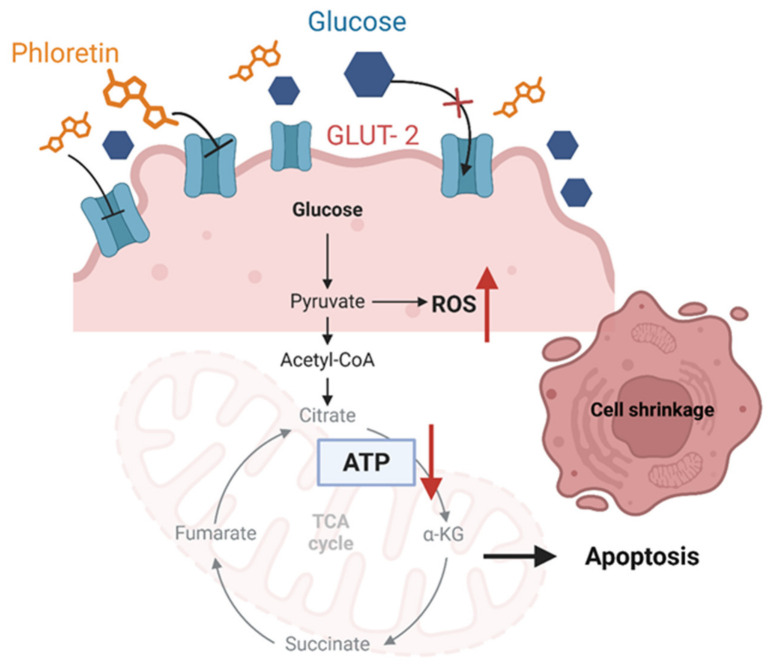
An expected mechanism of phloretin to inhibit HCC. Phloretin is hypothesized to inhibit HCC by inducing apoptosis in liver cancer cells. Its primary mechanism involves suppressing glucose uptake by directing inhibiting the type II glucose transporter (GLUT-2). This leads to reduced ATP production, triggering mitochondrial membrane potential breakdown and activating apoptotic signaling cascades, ultimately inducing apoptosis in HCC cells. (ATP, adenosine triphosphate; HCC, hepatocellular carcinoma). Created in BioRender. Juncheed, K. (2026) https://BioRender.com/lyt4toy (accessed on 15 May 2026).

Based on our observations, we propose an expected mechanism through which phloretin-induced glucose deprivation triggers apoptosis in HCC cells. The resultant reduction in glucose uptake is hypothesized to induce significant metabolic stress, initiating a cascade of cytotoxic intracellular events [62]. This metabolic perturbation likely leads to an energy crisis characterized by decreased adenosine triphosphate (ATP) synthesis, which is critical for impeding cancer cell proliferation and replicative capacity [37,63]. Beyond energy depletion, glucose transport inhibition profoundly impacts cellular redox homeostasis, potentially inducing oxidative stress [64]. While cancer cells typically maintain balanced levels of reactive oxygen species (ROS) through glucose metabolism, the disruption of this transport is expected to elevate intracellular ROS to damaging levels, affecting DNA and other vital cellular components [65,66,67]. Furthermore, the disruption of glucose transport and metabolism is intrinsically linked to the initiation of apoptosis, particularly through the mitochondrial death pathway [60,68]. This pathway can be triggered by ATP depletion and oxidative stress, or via mechanisms involving hypoxia-inducible involving hypoxia-inducible factor-1a and p53-associated pathways [69]. Phloretin has previously been shown to induce apoptosis in hepatoma cells by suppressing glucose transport, leading to mitochondrial dysfunction [61]. An expected mechanism of phloretin on glucose uptake and its subsequent induction of lethal metabolic and organelle stress, as comprehensively illustrated in Figure 10. Although biochemical markers such as ATP levels, ROS production, and caspase activation were not directly quantified in this study, the expected mechanistic role of phloretin is supported by distinct morphological alterations, including cellular shrinkage and membrane blebbing as illustrated in Figure 7. To definitively confirm this proposed mechanism, further investigation involving direct measurements of ROS probes, ATP assays, and mitochondrial membrane potential (ΔΨm) is required to fully validate these downstream metabolic effects.

### 4.4. Outlook and Future Direction

The findings from using phloretin as a GLUT-2 inhibitor in an HCC cellular model (HepG2 cell line), compared with a normal liver model (THLE-2 cell line), present a promising outlook for therapeutic strategies. The observed selective toxicity of phloretin towards HCC cells is largely attributable to their heightened dependence on GLUT-2 for glucose uptake, suggesting a significant potential for targeted therapy with minimal off-target effects on healthy hepatocytes. By effectively “starving” cancer cells through GLUT-2 inhibition, phloretin initiates a cascade of metabolic stress, including ATP depletion [37,70] and oxidative stress [64], ultimately leading to apoptosis and reduced proliferation [61,70]. This differential sensitivity provides a strong rationale for further investigating phloretin, or its derivatives, as potent metabolic-targeting agents to overcome chemoresistance and improve treatment outcomes in HCC [45]. The ability to preferentially induce apoptosis in malignant cells while sparing normal liver cells is a key advantage that warrants advanced preclinical and ultimately clinical exploration [17,61]. Therefore, our findings highlight phloretin as both a proof-of-concept therapeutic lead and a valuable reference inhibitor to inform the rational design of next-generation GLUT-2–targeted agents for HCC.

Despite this promising outlook, some inherent limitations should be acknowledged when extrapolating results from both HCC and normal liver cellular models to *in vivo* and clinical settings. First, our two-dimensional cellular models fundamentally lack the complex three-dimensional (3D) architecture, crucial cell–cell interactions, stromal components, and critical microenvironmental factors (e.g., hypoxia, nutrient gradients, and extracellular matrix) that are intrinsically present in both healthy and cancerous living liver [71]. These multifaceted biological and physical factors can significantly influence drug penetration, metabolism, and therapeutic efficacy. Consequently, future research should comprehensively address these limitations by transitioning to more physiologically relevant *in vitro* models, including 3D spheroids and organoids, and by advancing to *in vivo* animal models. These steps are essential to rigorously evaluate phloretin’s pharmacokinetics, bioavailability, systemic toxicity, and overall therapeutic efficacy, as well as to investigate potential resistance mechanisms and optimal combination therapy strategies [37,72]. Furthermore, to fully validate the anticancer properties and delineate the precise mechanism of inhibition, additional *in vitro* investigations are imperative. These should encompass methodologies, including double staining with acridine orange and propidium iodide to robustly assess morphological apoptotic cell induction and thiobarbituric acid reactive substance assays to confirm oxidative stress levels. Lastly, to gain a deeper understanding of the molecular interactions, molecular dynamics simulations would provide invaluable insights into the dynamic binding characteristics and stability of phloretin–GLUT2 interactions over time.

While this study provides a robust molecular rationale for the interaction between phloretin and GLUT-2, we acknowledge that experimental validation via site-directed mutagenesis or CRISPR-Cas9 gene editing remains the gold standard for definitively confirming specific binding sites. Although these complex genetic manipulations were outside the initial scope of this research, which focused on characterizing phloretin as a natural molecular scaffold, they represent a critical next step. Future investigations should utilize mutagenesis to verify the functional role of the specific amino acid residues identified in our docking model, such as GLN193, GLN315, and PHE24. Such studies will provide definitive proof of the binding mechanism and facilitate the transition from a prominent molecular template to a validated therapeutic lead for targeting glucose metabolism in hepatocellular carcinoma.

## 5. Conclusions

This study demonstrates that phloretin, a natural polyphenol, modulates glucose transport in HCC cells through interactions with the GLUT-2 transporter. *In vitro* assessments revealed that phloretin effectively reduced the metabolic activity of HepG2 cells in a dose- and time-dependent manner, while also suppressing 2-NBDG uptake. This finding highlights the metabolic dependency of these malignant cells. Molecular docking provided computational support for these observations, suggesting a competitive binding mode where phloretin interacts with key residues within the GLUT-2 substrate cavity through stabilized aromatic and hydrogen bond interactions. Collectively, these findings position phloretin as a prominent molecular scaffold and a valuable experimental reference for GLUT-2-oriented metabolic studies. While phloretin provides a significant starting point for understanding glucose transport inhibition, its development into a therapeutic lead requires further validation of its isoform selectivity and *in vivo* efficacy. By integrating experimental and computational evidence, this study establishes a foundation for the rational design of more potent GLUT-2 inhibitors and for future exploration of metabolic targeting strategies in HCC.

## Figures and Tables

**Table 1:** Summary of ligand-GLUT interactions identified from Class I GLUT isoforms docking analysis.

Ligand (Substrate/Inhibitor)	Protein Target	Free Binding Energy (kcal/mol)	Number of Interactions		
			H-Bond	π–Interaction	Steric Hindrance
Glucose	GLUT-1	−4.37	7	0	0
	GLUT-2	−4.68	7	0	0
	GLUT-3	−4.38	5	0	1
	GLUT-4	−4.43	4	0	3
Phloretin	GLUT-1	−7.28	4	3	0
	GLUT-2	−6.96	5	5	0
	GLUT-3	−7.28	6	0	1
	GLUT-4	−7.48	5	3	0
Glutor	GLUT-1	−9.60	7 ^#^	7 **	1
	GLUT-2	−10.94	4 ^#^	4	0
	GLUT-3	−9.43	2 ^#^	5 **	0
	GLUT-4	−10.06	2 ^#^	7 *^,^**	1
Cytochalasin B	GLUT-1	−8.90	2	3	1
	GLUT-2	−9.69	2	3 **	0
	GLUT-3	−8.93	2	2	0
	GLUT-4	−8.96	3	2	1

Note: ^#^ Carbon hydrogen bond, * pi-sulfur ** pi-sigma. GLUT, glucose transporter.

## Data Availability

The datasets generated and/or analyzed during the current study are not publicly available due to the need to keep them confidential, but are available from the corresponding author upon request.

## Supplementary Materials

The following supporting information can be downloaded at: https://www.mdpi.com/article/10.3390/biomedicines14051166/s1, Figure S1: Time-dependent 2-NBDG uptake in HepG2 and THLE-2 cells; Figure S2: Dose-response profiles of phloretin-treated cells at 24 h; Figure S3: Comparative interaction analysis of glucose and phloretin within the GLUT-2 binding pocket.

## Abbreviations

The following abbreviations are used in this manuscript:

2-NBDG	2-(N-(7-Nitrobenz-2-oxa-1,3-diazol-4-yl)Amino)-2-Deoxyglucose
ATP	Adenosine triphosphate
FI	Fluorescence intensity
GLUT	Glucose transporter
GLUT-1	Glucose transporter type 1
GLUT-2	Glucose transporter type 2
GLUT-3	Glucose transporter type 3
GLUT-4	Glucose transporter type 4
HCC	Hepatocellular carcinoma
IC_50_	Half-maximal inhibitory concentration
MTT	3-(4,5-Dimethylthiazol-2-yl)-2,5-diphenyltetrazolium bromide
CID	Compound Identifier (PubChem)
ROS	Reactive oxygen species
