# Supplementary material for: Development of Type II Glucose Transporter Inhibitors: Phloretin as a GLUT-2 Screening Template from In Silico Modeling to In Vitro Assessment
Source: Biomedicines. 2026 May 21;14(5):1166. doi: 10.3390/biomedicines14051166 (PMC13204967; doi:10.3390/biomedicines14051166)
Supplement: Supplementary file 1 [file biomedicines-14-01166-s001.zip › biomedicines-4264227-supplementary.pdf]

Supplementary Material

Article: Development of Type II Glucose Transporter Inhibitors**:** Phloretin as a GLUT-**2** Screening Template from *In Silico* Modeling to *In Vitro* Assessment

Worarat Boonpech [1,2], Pemikar Srifa [2,3], Dhassida Sooksawat [4,5], Praopim Limsakul [4,5], Jirakrit Saetang [6], Varomyalin Tipmanee [2,7], Krit Charupanit [1,2], Chaitong Churuangsuk [7,8], and Kantida Juncheed [1,2,7,*]

[1]Institute of Biomedical Engineering, Faculty of Medicine, Prince of Songkla University, Hat Yai, Songkhla, 90110, Thailand
[2]Department of Biomedical Sciences and Biomedical Engineering, Faculty of Medicine, Prince of Songkla University, Hat Yai, Songkhla, 90110, Thailand
[3]Translational Medicine Research Center (TMRC), Department of Biomedical Sciences and Biomedical Engineering, Faculty of Medicine, Prince of Songkla University, Hat Yai, Songkhla, 90110, Thailand
[4]Division of Physical Science, Faculty of Science, Prince of Songkla University, Hat Yai, Songkhla, 90110, Thailand
[5]Center of Excellence for Trace Analysis and Biosensor, Prince of Songkla University, Hat Yai, Songkhla, 90110, Thailand
[6]International Center of Excellence in Seafood Science and Innovation, Faculty of Agro-Industry, Prince of Songkla University, Hat Yai, Songkhla, 90110, Thailand
[7]Center of Biological Activities Testing, Department of Biomedical Sciences and Biomedical Engineering, Faculty of Medicine, Prince of Songkla University, Hat Yai, Songkhla, 90110, Thailand
[8]Clinical Nutrition and Obesity Medicine Unit, Department of Internal Medicine, Faculty of Medicine, Prince of Songkla University, Hat Yai, Songkhla 90110, Thailand

Corresponding and requests for material should be addressed to: kantida.j@psu.ac.th

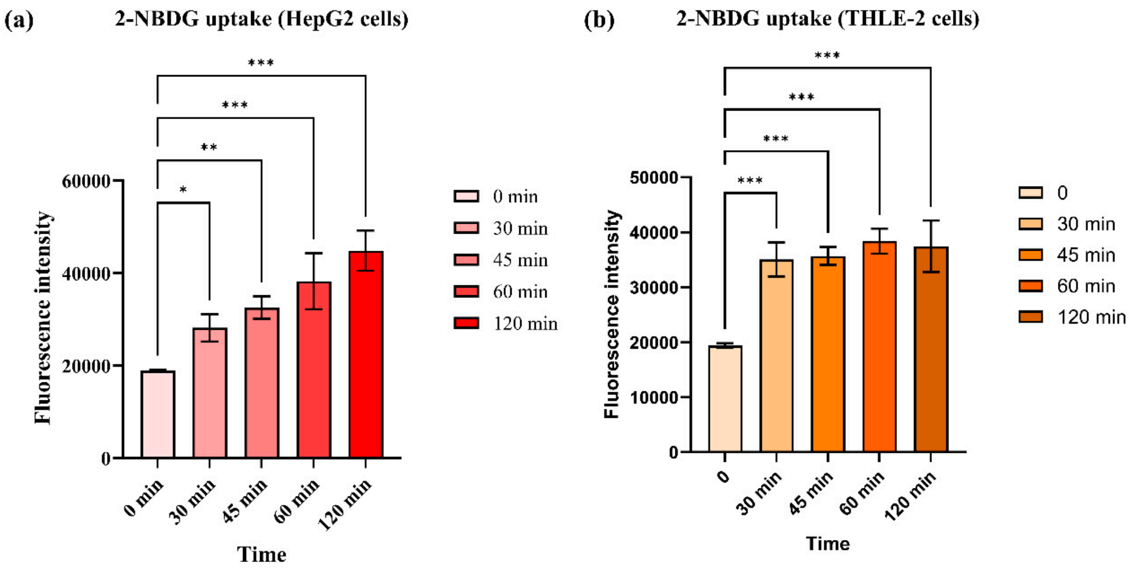

Figure S*1* Time-dependent 2-NBDG uptake in HepG2 and THLE-2 cells. Fluorescence intensity was measured at 0, 30, 45, 60, and 120 minutes. Data are expressed as mean ± SD (n = 3). Statistical analysis was performed using one-way ANOVA followed by Dunnett's multiple comparisons test versus the 0 min control (*$p < 0.05$, **$p < 0.01$, ***$p < 0.001$).

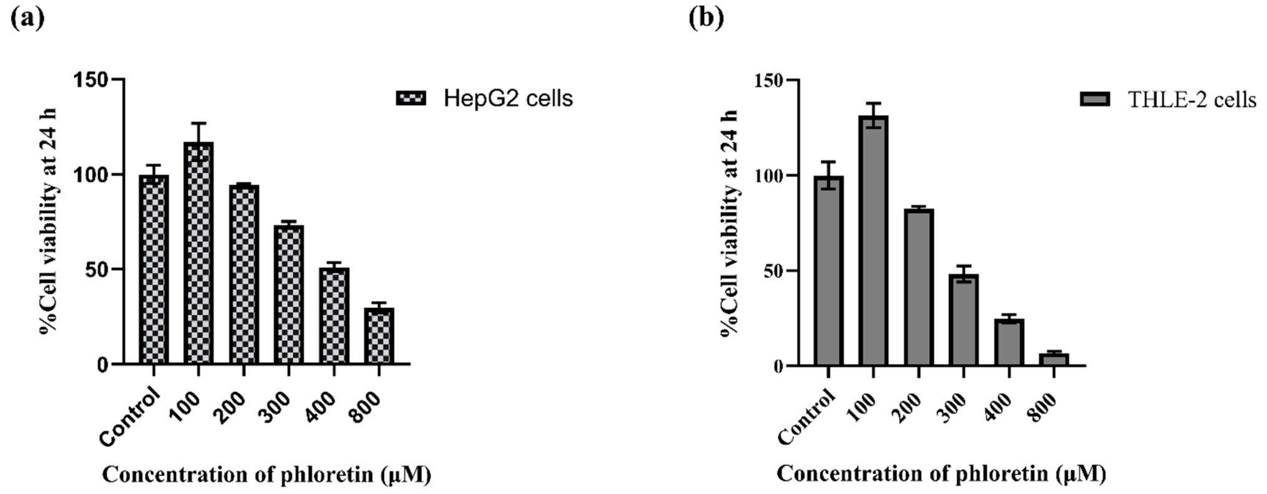

Figure S2 Dose-response profiles of phloretin-treated cells at 24 h. The percentage of cellular metabolic activity was determined via MTT assay for (a) HepG2 cells and (b) THLE-2 cells following exposure to various concentrations of phloretin (100, 200, 300, 400, and 800 μM). Data are presented as mean ± SD (n = 3).

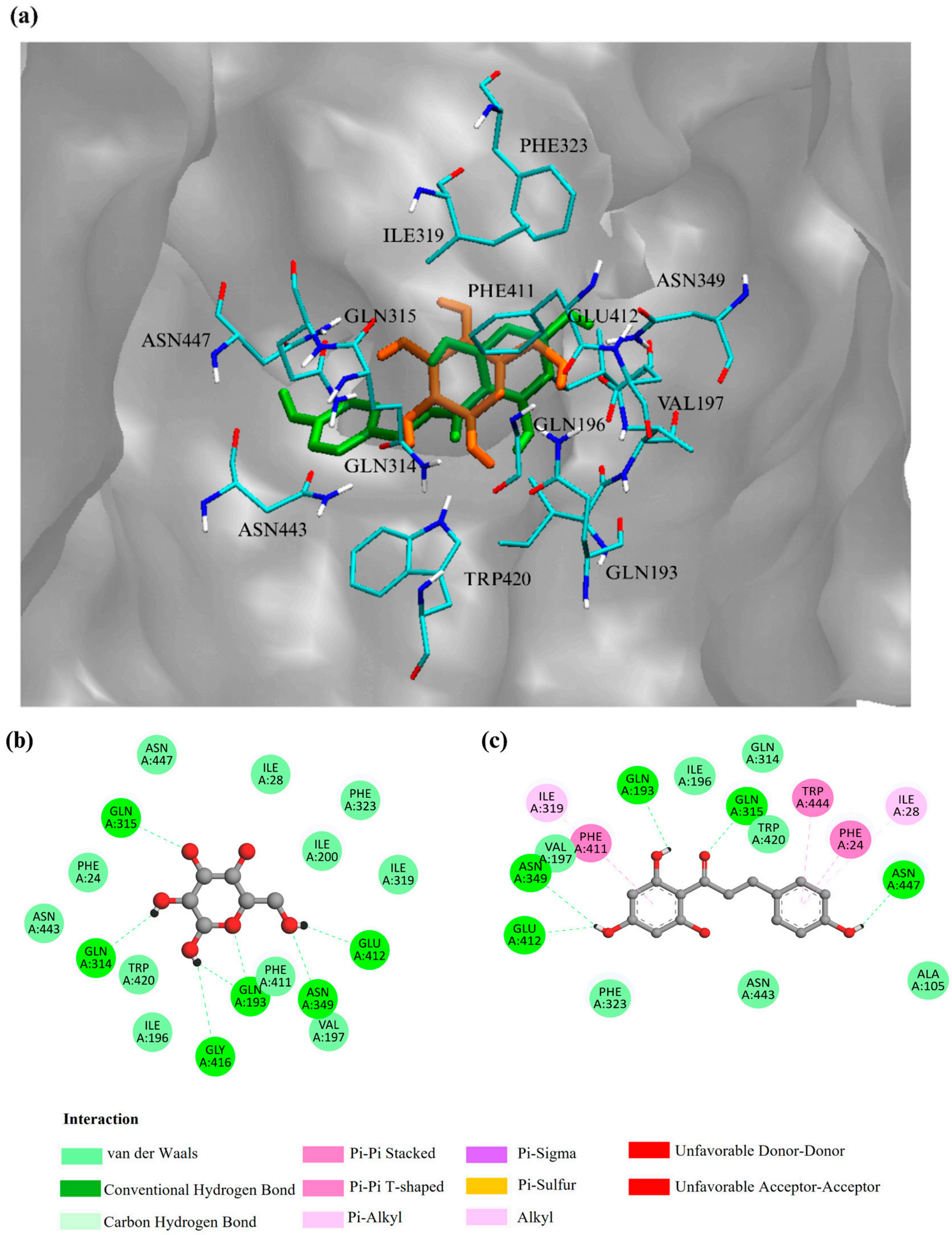

Figure S3 Comparative interaction analysis of glucose and phloretin within the GLUT-2 binding pocket. (a) Three-dimensional docking poses of glucose (green) and phloretin (orange) within the GLUT-2 cavity and surrounding interacting residues. (b) Two-dimensional interaction map of glucose with GLUT-2. (c) Two-dimensional interaction map of phloretin with GLUT-2, highlighting hydrogen bonding and π-mediated interactions. Structural visualization was generated using VMD and Discovery Studio Visualizer.
